# Extracorporeal membrane oxygenation for pheochromocytoma-induced cardiogenic shock

**DOI:** 10.1186/s13613-016-0219-4

**Published:** 2016-11-28

**Authors:** Guillaume Hekimian, Fatima Kharcha, Nicolas Bréchot, Matthieu Schmidt, Cécile Ghander, Guillaume Lebreton, Xavier Girerd, Christophe Tresallet, Jean-Louis Trouillet, Pascal Leprince, Jean Chastre, Alain Combes, Charles-Edouard Luyt

**Affiliations:** 1 Intensive Care Unit, Institute of Cardiology, Groupe Hospitalier Pitié–Salpêtrière, Assistance Publique-Hôpitaux de Paris, Paris, France; 2 UPMC Université Paris 06, INSERM, UMRS_1166-ICAN Institute of Cardiometabolism and Nutrition, Paris, France; 3 Institute of Endocrinology, Pierre et Marie Curie Sorbonne Université, APHP, Groupe Hospitalier Pitié–Salpêtrière, Paris, France; 4 Department of Cardiac and Thoracic Surgery, Pierre et Marie Curie Sorbonne Université, APHP, Groupe Hospitalier Pitié–Salpêtrière, Paris, France; 5 Department of General and Endocrine Surgery, Pierre et Marie Curie Sorbonne Université, APHP, Groupe Hospitalier Pitié–Salpêtrière, Paris, France

## Abstract

**Background:**

Pheochromocytoma, a rare catecholamine-producing tumor, might provoke stress-induced Takotsubo-like cardiomyopathy and severe cardiogenic shock. Because venoarterial-extracorporeal membrane oxygenation (VA-ECMO) rescue of pheochromocytoma-induced refractory cardiogenic shock has rarely been reported, we reviewed our ICU patients’ presentations and outcomes.

**Methods:**

All pheochromocytoma-induced refractory cardiogenic shock cases managed with VA-ECMO (January 2007–March 2015) were prospectively included and reviewed. We also performed a systematic review on this topic.

**Results:**

Nine patients (7 women, 2 men; 31–51 [median, 43 (IQR 36–49) years old]) were included; none had a previously known pheochromocytoma. Six of them had medical histories suggestive of the diagnosis: palpitations and headaches for several months for four, multiple endocrine neoplasia syndrome type 1 for one and recurrent Takotsubo disease for one; at hospital admission, all were hypertensive despite cardiogenic shock. Three others had an identified surgical triggering factor. All nine patients rapidly developed refractory cardiogenic shock with very severe left ventricular (LV) impairment (LV ejection-fraction range 5–20%; LV outflow-tract velocity–time integral range 3–8 cm). Seven patients’ abdominal computed tomography scans showed pheochromocytoma-suggestive adrenal gland tumors (no scan during ICU stay for 2). Despite VA-ECMO implantation, three patients died of refractory multiple organ failure. For the six others, myocardial function improved and ECMO was removed 3–7 days post-implantation; α- and β-blockers were progressively introduced. Five survivors underwent pheochromocytoma excision 3 weeks–4 months post-ICU discharge, with satisfactory outcomes. One patient, whose pheochromocytoma was diagnosed 1 year after the index event, underwent uneventful surgical adrenalectomy. Systematic review retrieved 40 cases of pheochromocytoma-induced cardiogenic shock requiring mechanical support (mostly ECMO), with a mortality rate of 7%. Pheochromocytoma was removed surgically after mechanical support weaning in 31 patients and during mechanical support in 5. Four were not operated.

**Conclusions:**

Pheochromocytoma is a rare but reversible cause of cardiogenic shock amenable to VA-ECMO rescue. Adrenal gland imaging should be obtained for all patients with unexplained cardiogenic shock. Lastly, it might be safer to perform adrenalectomy several weeks after the initial catastrophic presentation, once recovery of LV systolic function is complete.

## Background

Pheochromocytoma, called paraganglioma when extra-adrenal, is a rare catecholamine-secreting neuroendocrine tumor arising from chromaffin cells in the adrenal medulla. Its usual clinical picture combines persistent or paroxysmal hypertension, palpitations, headache, diaphoresis, tremors and/or anxiety. However, life-threatening complications, such as Takotsubo-like cardiomyopathy and cardiogenic shock, have been described and are often fatal. In this setting, venoarterial-extracorporeal membrane oxygenation (VA-ECMO) might be the only way to prevent death, but only a few cases of ECMO-managed pheochromocytoma-induced cardiogenic shock have been published.

## Methods

We reviewed the presentations and outcomes of nine patients admitted to our ICU for pheochromocytoma-induced refractory cardiogenic shock rescued by VA-ECMO between January 2007 and March 2015 (Table 1). For our literature review, the MEDLINE database was searched using “pheochromocytoma” and “cardiogenic shock” as key words. Articles were screened and those reporting cardiogenic shock requiring mechanical circulatory support (except single intra-aortic balloon pump) were selected.

**Table 1:** Clinical and biological characteristics of the nine patients

Characteristic	Case 1	Case 2	Case 3	Case 4	Case 5	Case 6	Case 7	Case 8	Case 9
Sex	Male	Female	Female	Female	Female	Female	Female	Male	Female
Age (years)	41	44	36	45	43	51	49	31	37
Medical history									
Palpitations/headaches/sweating/hypertension	Yes	Yes	Yes					Yes	
Other			Type 1 neurofibromatosis		Takotsubo				
Blood pressure at admission (mmHg)	160/120	180/108	157/107	130/110	140/100	130/85	141/107	160/100	129/99
Pre-ECMO LVEF (%)	5	20	15	20	30	20	20	30	20
Maximum troponin I (ng/L)	2200	34,000	43,000	15,000	2000	18,000	237	4994	2083
NT pro-BNP at admission (ng/L)			18,363		25,282	6363	18,471		9264
ECMO duration (days)	4	1	6	7	3	3	2	7	4
Urinary metanephrine (*N* < 340 µg/24 h)	ND	ND	ND	ND	1894	7192	8738	2862	2582
Urinary normetanephrine (*N* < 440 µg/24 h)	ND	ND	ND	ND	4973	3650	11,910	11,268	9254
Pheochromocytoma size (mm), side	78 × 75 × 70, right	60 mm, left	ND, autopsy	29, left	70, left	103 × 95 × 75, right	57 × 47, right	59 × 38, left	88 × 74×91, left

*ECMO* extracorporeal membrane oxygenation, *LVEF* left ventricle ejection fraction, *N* normal value, *ND* not done

## Results

### Case reports

#### Case 1

A 41-year-old man consulted the emergency department (ED) for acute abdominal pain with nausea and vomiting. He had a 1-year history of hypertension and stated having had palpitations and headaches for several years. At admission, his blood pressure was 160/120 mmHg; physical examination and chest X-ray were consistent with severe pulmonary edema. His electrocardiogram indicated sinus tachycardia and ST-segment elevation in leads V1–V3. Transthoracic echocardiography (TTE) showed very severe left myocardial impairment with left ventricular ejection fraction (LVEF) at 5%. Dobutamine then epinephrine infusion obtained no improvement. Under VA-ECMO implanted before he was transferred to our ICU, he rapidly recovered cardiac function, allowing ECMO weaning on day 4. Post-weaning, he developed severe hypertension that required nicardipine infusion. Abdominal ultrasonography visualized a 7-cm-diameter lesion in the right adrenal gland, confirmed by computed tomography (CT). Four days after ECMO weaning, while under nicardipine infusion, labetalol and ramipril, he developed malignant hypertension (blood pressure at 270/120 mmHg) and cardiogenic shock complicated by cardiac arrest. VA-ECMO was re-implanted, but he died of refractory multiorgan failure. Autopsy confirmed the pheochromocytoma diagnosis.

#### Case 2

A 44-year-old woman underwent elective hysteroscopic resection of a bleeding uterine fibroid. Her medical history included migraines, sweating episodes, appendicular peritonitis in childhood and uterine fibroids. While in the recovery room, she became hypertensive (blood pressure 180/108 mmHg) and polypneic, with clinically and radiographically diagnosed pulmonary edema. TTE-estimated LVEF was 20%. Despite nitrates and diuretics, her clinical status abruptly deteriorated with cardiogenic shock, renal and hepatic failures, and hyperlactatemia (9 mmol/L). She was intubated, epinephrine infusion was started and progressively increased to 4 mg/h, and our ECMO mobile team implanted VA-ECMO. Post-implantation, her severe hypertension (mean blood pressure at 150 mmHg) required antihypertensive therapy. Abdominal CT revealed a 6-cm tumor in the right adrenal gland. Her hemodynamic status worsened under VA-ECMO with major capillary leak syndrome requiring vascular filling; she died several hours later.

#### Case 3

After 1 week of ineffective antibiotics, a 36-year-old woman was referred to her local ED for persistent febrile headaches, cough and vomiting. Her history consisted of type 1 neurofibromatosis, headaches and palpitations. At admission, her blood pressure was 157/107 mmHg and her heart rate 162 beats/min. Suddenly, she developed massive pulmonary edema with cardiogenic shock requiring mechanical ventilation and dobutamine infusion. TTE revealed severe global hypokinesia with LVEF at 20%. She developed multiorgan failure, and our ECMO mobile team implanted VA-ECMO. On day 4, she suffered a massive ischemic stroke, cerebral herniation and cerebral death. During her stay, abdominal ultrasonography found a left adrenal tumor; autopsy confirmed a left adrenal pheochromocytoma.

#### Case 4

After 2 weeks of antibiotics and corticosteroids for bronchitis, a 45-year-old woman went to her local ED for acute chest pain. She had no remarkable medical history. At admission, BP was 130/110 mmHg, her heart rate was 86 beats/min, and oxygen saturation was 98% breathing ambient air. She rapidly developed signs of respiratory and hemodynamic failure, and mechanical ventilation was started. Her electrocardiogram indicated sinus tachycardia and ST-segment elevation in leads V2–V3. TTE revealed severe LV dysfunction with LVEF at 20%. Refractory electrical storm ensued, requiring VA-ECMO implantation by the ECMO mobile team after 45 min of cardiopulmonary resuscitation. Peripheral ECMO was switched to central ECMO during her ICU stay. Cardiac biopsies taken during the switch contained fibrosis and lymphocytic infiltration without necrosis, suggestive of borderline myocarditis. Heart function recovered and ECMO was removed 7 days post-implantation. After 5 weeks in ICU complicated by ischemic stroke, she was discharged from the hospital. Several months later, a CT scan for abdominal pain visualized an adrenal mass, whose radiologic appearance suggested pheochromocytoma. She underwent laparoscopic adrenalectomy under stable hemodynamic conditions; histologic examination of the mass confirmed the pheochromocytoma diagnosis.

#### Case 5

A 43-year-old woman went to her ED for acute chest pain, palpitations, dyspnea and vomiting a few hours after dental care. She had been hospitalized 8 months earlier for acute coronary syndrome with normal coronary angiography after mammoplasty, and 4 months ago for Takotsubo syndrome following dental care. At admission, her BP was 140/100 mmHg and pulse rate 97/min. Acute respiratory distress required immediate mechanical ventilation. Her electrocardiogram revealed negative T waves on lateral leads. Troponin I was at 2 µg/L. TTE found severe LV systolic dysfunction (LVEF at 20%), with apical ballooning. Despite infused dobutamine and epinephrine (gradually increased to 15 mg/h), circulatory failure worsened and femoral–femoral VA-ECMO was implanted. Within few hours, the patient’s hemodynamics improved and, after discontinuing catecholamine infusion, a hypertensive crisis occurred. Abdominal ultrasonography discovered a 7-cm left adrenal lesion confirmed by CT. ECMO was removed on day 3 with full recovery of heart systolic function; α- and β-blockers (prazosin and atenolol) were started. Six weeks after admission, she underwent uncomplicated adrenalectomy; histologic examination of the mass confirmed the pheochromocytoma diagnosis.

#### Case 6

A 51-year-old woman, with no remarkable medical history, underwent elective abdominal hernia repair. During surgery, she developed hypertensive cardiogenic pulmonary edema. TTE revealed Takotsubo cardiomyopathy, with LV apical akinesis and LVEF at 25%. Her electrocardiogram indicated ST-segment depression on lateral leads. Inotropic support was ineffective, and VA-ECMO was implanted. Abdominal CT showed a 10-cm-diameter lesion in the right adrenal gland with central necrosis. Elevated urinary metanephrine and normetanephrine, and scintigraphy with metaiodobenzylguanidine confirmed the pheochromocytoma diagnosis. Prazosin (α-blocker) was initiated, and she was weaned off ECMO on day 3. During hospitalization, she developed numerous hypertensive crises that were controlled medically. The lesion/mass was successfully excised 6 weeks post-admission. Histology of the surgical specimen confirmed necrotic pheochromocytoma. The patient was well at 3-year follow-up.

#### Case 7

A 49-year-old woman, with no remarkable medical history, had fever, cough and myalgia of 2-week duration. She was referred to the ED for acute dyspnea and circulatory failure. At admission, her blood pressure was 120/100 mmHg, pulse 137/min and severe cardiogenic pulmonary edema required rapid intubation. TTE-measured LVEF was at 20%. Antibiotics and oseltamivir were started for suspected fulminant myocarditis. Despite maximal inotropic support, adequate perfusion could not be achieved and femoral–femoral VA-ECMO was implanted, complicated a few hours later by acute right inferior limb ischemia with compartment syndrome. On day 2, partial hemodynamic improvement allowed ECMO removal and right limb aponeurotomy. Abdominal ultrasonography visualized a large right adrenal mass. LV systolic function recovered fully. Antihypertensive medications (prazosin and β-blockers) were started. Right adrenalectomy was performed 6 weeks later; histologic examination of the mass confirmed the pheochromocytoma diagnosis.

#### Case 8

A 31-year-old man consulted his local ED for palpitations, and headaches associated with chest and abdominal pain. During the preceding year, he had experienced episodic headaches, sweating and palpitations. At physical examination BP was 160/100 mmHg, pulse 160/min, temperature 38.5 °C. His electrocardiogram indicated ST-segment depression in inferior–lateral leads, and troponin I was at 4994 ng/L. Emergency coronary artery angiography was normal. His respiratory and hemodynamic status worsened, necessitating mechanical ventilation and hemodynamic support. TTE showed severe LV hypokinesia with LVEF at 30%. VA-ECMO was implanted for refractory cardiogenic shock. Abdominal CT discovered a 5-cm left adrenal mass. Prazosin was started and controlled hemodynamic variations. ECMO was removed 7 days post-implantation. The patient underwent surgery 6 weeks later and was discharged in good condition.

#### Case 9

Two days after the onset of flu-like symptoms, a 47-year-old woman with a history of morbid obesity and depression went to the ED for abdominal pain, vomiting and myalgia. Physical examination found temperature at 38.4 °C, BP 129/99 mmHg, pulse 138/min, respiratory rate 24/min and initial oxygen saturation 97% breathing ambient air, but the rapid deterioration of her respiratory and hemodynamic status required oxygen therapy and mechanical ventilation. Her echocardiography revealed severe LV dysfunction, with apical thrombosis. Troponin I was at 2083 ng/L. Despite inotropic support, her hemodynamics worsened and VA-ECMO was implanted. Hemodynamics quickly improved, and ECMO was removed on day 4. She suffered an embolic stroke that caused left hemiplegia. Abdominal CT showed a multinodular formation near the left adrenal gland, whose enhancement and density were compatible with a paraganglioma. She was started on α- and β-blockers and discharged to a neurologic rehabilitation center. Her tumor was excised 4 months later; histology confirmed a voluminous paraganglioma.

#### Literature review

We conducted a systematic MEDLINE database literature review through the PubMed search engine, between 2000 and 2016, using the following criteria: pheochromocytoma and cardiogenic shock, pheochromocytoma and ECMO, pheochromocytoma and mechanical circulatory support, Takotsubo and cardiogenic shock, adrenergic cardiomyopathy and cardiogenic shock. We also searched the references of identified studies. Observational studies and case series reporting on adult patients having required mechanical circulatory support for pheochromocytoma-induced cardiogenic shock were eligible. Studies on children or newborns, those without any outcome information, were excluded.

Two authors (G.H. and N.B.) independently reviewed the retrieved abstracts and assessed eligibility. A third author (C.E.L.) determined eligibility in the case of disagreement.

The following data were extracted: study design, participants’ characteristics (including echocardiographic data), type of mechanical circulatory support, outcome data (hospital mortality).

Forty cases of patients suffering from pheochromocytoma-induced cardiogenic shock requiring mechanical circulatory support (except use of intra-aortic balloon pump alone) were reported in the literature (Table 2). Twenty (50%) were women, and their median age was 40 years (IQR 31–49 years). Left ventricular dysfunction was very severe in all cases with a median LVEF of 15% (IQR 10–20%). ECMO was used in 35 patients, 2 patients had Impella device, 1 had Tandem Heart device, 2 had cardiopulmonary bypass to facilitate pheochromocytoma surgery, and 4 patients had long-term mechanical circulatory support. Median duration of mechanical circulatory support was 4 days (IQR 1.5–6.5 days). Pheochromocytoma was removed surgically after mechanical circulatory support weaning in 29 patients and while on mechanical circulatory support in 7. Two patients died before surgery, one patient declined, and the timing of surgery was not reported in the last case. The median ICU-admission-to-surgery interval was 23 days (IQR 1–46 days). Thirty-seven of the 40 (93%) reported cases survived.

**Table 2:** Case reports of pheochromocytoma-induced cardiogenic shock requiring mechanical circulatory support

First author	Year	Patients with cardiac assist device, *n*	Mean age (years)	Sex	Initial LVEF (%)	Mechanical circulatory support; duration (days)	ICU-admission -to-surgery interval (days)	Hospital survival
Takagi [17]	2000	1	52	M	9	ECMO and IABP; 3	NR, after ECMO weaning	Yes
Grinda [18]	2006	1	49	M	12	ECMO, then LVAD; 4, then 70, respectively	4	Yes
Ouchikhe [19]	2006	1	23	M	20	ECMO, then Thoratec BiVAD; 84	93	Yes
Kim [20]	2007	1	47	M	23	ECMO and IABP; 10	15	Yes
Chao [21]	2008	1	42	M	20	ECMO and IABP; 7	18	Yes
Zegdi [22]	2008	1	51	F	30	ECMO; 6	30	Yes
Huang [23]	2008	3	25	F	NR	ECMO and IABP; 4	NR, after ECMO weaning	Yes
			42	M	NR	ECMO and IABP; 7	NR, after ECMO weaning	Yes
			41	F	<NR	ECMO and IABP; 2	NR, after ECMO weaning	Yes
Grasselli [24]	2008	1	47	F	30	ECMO; 4	7	Yes
Suh [25]	2008	1	43	M	27	ECMO and IABP; 4	56	Yes
Wu [26]	2008	2	42	M	34	ECMO and IABP; 8	21	Yes
			40	M	30	ECMO and IABP; 4	12	Yes
Newton [27]	2008	1	45	M	10	CBP to facilitate pheochromocytoma surgery; 1	1	Yes
Park [28]	2009	1	41	M	20	ECMO and IABP; 2	30	Yes
Westaby [12]	2009	1	27	M	15	CBP and adrenalectomy, then LVAD; 6	1	Yes
Raikhelkar [29]	2010	1	38	M	5	Thoratec BiVAD; 4	24	Yes
Xeridat [25]	2011	1	16	F	20	ECMO; 6	21	Yes
Ritter [30]	2011	1	49	F	10	ECMO; 8	8	Yes
Nakajima [31]	2011	1	33	F	19	ECMO and IABP; 4	120	Yes
Contargyris [32]	2012	1	20	M	20	ECMO; 2	30	Yes
Sojod [33]	2012	1	37	F	15	ECMO; 11	21	Yes
Sheinberg [34]	2012	1	45	F	5	ECMO and IABP; 3	56	No
Noorani [35]	2012	1	39	F	NR	Central ECMO; 5	3	Yes
Banfi [36]	2012	1	20	F	20	Central ECMO; 6	120	Yes
Kaese [37]	2013	1	43	M	NR	ECMO; 9	NR, after ECMO weaning	Yes
Law [38]	2013	1	23	F	5	ECMO; 1	NR, after ECMO weaning	Yes
Shawa [39]	2014	1	38	F	15	Tandem Heart; 1	26	Yes
Riester [1]	2015	2	18	F	NR	Impella; 2	No surgery	No
			25	F	NR	ECMO; NR	NR, after ECMO weaning	Yes
Chao [2]	2015	4, 2 with IABP^a^	25	F	NR	ECMO^a^	NR, after ECMO weaning	Yes
			52	M	NR	ECMO^a^	NR, after ECMO weaning	Yes
			40	M	NR	ECMO^a^	NR, after ECMO weaning	Yes
			65	M	NR	ECMO^a^	Refused surgery	Yes
Zhou [40]	2015	1	35	M	20	ECMO and IABP during cardiac paraganglioma resection; 16	Cardiac paraganglioma resection, date unknown	Yes
Flam [10]	2015	1	46	F	15	ECMO; 5	66	Yes
Vagner [41]	2015	1	55	F	15	Impella, then ECLS; 2	56	Yes
Spangenberg [14]	2015	1	29	F	10	ECMO and IABP; 1	No surgery	No
Kodama [9]	2016	1	37	F	10	ECMO; NR	NR, before ECMO weaning	Yes
Dang Van [42]	2016	1	57	M	10	ECMO, 7	NR, concomitant from the ECMO implantation	Yes

*IABP* intra-aortic balloon pump, *NR* not reported, *CPB* cardiopulmonary bypass, *LVAD* left ventricular assist device, *BiVAD* biventricular assist device, *ECLS* extracorporeal life support

^a^Chao et al. did not specify which two patients had ECMO and IABP and individual ECMO durations were not given

## Discussion

We described nine critical presentations of undiagnosed pheochromocytoma with refractory cardiogenic shock requiring VA-ECMO salvage therapy.

Many cardiac manifestations of pheochromocytoma have been reported, including Takotsubo-like cardiomyopathy, myocardial infarction, pulmonary edema and/or hypertensive crisis [1], but only rarely inaugural refractory cardiogenic shock requiring VA-ECMO (Table 2). This is, to our knowledge, the largest case series of pheochromocytoma-induced refractory cardiogenic shock. As previously reported [1, 2], the major clinical feature was the sequence of initial hypertensive crisis followed by hypotensive shock, probably caused by severe LV failure. Following ECMO implantation, all our patients suffered paroxysmal hypertension with wide and rapid blood pressure fluctuations.

Coronary angiography should be considered in every patient with unexplained cardiogenic shock, especially in cases of ST-segment deviation. It was performed in patient 8, and patient 5 had a normal coronary catheterization a few months ago. In our study, the other patients did not have this examination because three had a clinical presentation highly suggestive of myocarditis and four had clinical characteristics suggestive of pheochromocytoma with early diagnosis performed on abdominal imaging.

Cardiogenic shock is a rare but potentially lethal pheochromocytoma manifestation, and in our series, three of the nine patients died. For the six survivors, mechanical circulatory support was probably the only way to prevent death. Because ECMO allows quick and easy percutaneous insertion of cannulae, full circulatory support and improved tissue oxygenation in situations of cardiogenic shock combined with severe pulmonary edema [3, 4], it was preferred over other assisted support options for all these patients. Pertinently, cardiac dysfunction reversibility in these pheochromocytoma-induced cardiomyopathies is well established; all six survivors were all weaned off ECMO within a week and recovered normal myocardial systolic function.

Catecholamine-mediated cardiomyopathy is not completely understood. Proposed mechanisms include coronary artery or microvascular spasms leading to focal myocardial necrosis [5], direct cellular toxicity through increased intracellular calcium concentrations [6] or damage induced by reactive oxygen species [7], and myocardial stunning due to receptor desensitization and/or downregulation [8]. When obtained, endomyocardial biopsies contained nonspecific contraction band necrosis [9]. Our patient 4’s suspected myocarditis biopsy contained lymphocytic infiltration and focal fibrosis, without necrosis.

In reported cases [10] and our experience, once the initial critical context has been controlled, heart failure is fully reversible within a few days, suggesting stunning or metabolic anomaly rather than myocardial necrosis.

Medical management of pheochromocytoma-induced cardiogenic shock raises some concerns. Various drugs, including adrenaline, noradrenaline, dobutamine, vasopressin and levosimendan, were used in case reports. Because exposure to elevated epinephrine levels may engender cardiac dysfunction and sympathetic receptor downregulation, catecholamines usually used to treat cardiogenic shock could be less effective or even exacerbate pheochromocytoma-induced cardiogenic shock. Some authors proposed alternative agents that do not act via adrenergic receptors, such as calcium-sensitizing agents or phosphodiesterase III inhibitors, but no available evidence supports those decisions [11, 12].

Cardiogenic shock was the first main manifestation of undiagnosed pheochromocytoma for most of our patients, but some clinical manifestations could help guide the diagnosis. The major characteristic was hypertension at admission contrasting with evidence of circulatory failure. Symptoms suggestive of pheochromocytoma, e.g., headaches, sweating and palpitations, were retrospectively found in four patients, one had type 1 neurofibromatosis, and one had experienced two Takotsubo cardiomyopathy episodes. During recovery, four of them developed severe paroxysmal hypertension, also evocative of pheochromocytoma.

Precipitating circumstances were identified for some patients. Three relatively asymptomatic patients’ previously undiagnosed pheochromocytomas were unmasked by elective surgery, manifesting as peri- or postoperative hypertensive crises and subsequent cardiogenic shock. Johnston et al. described two similar patients with hypertensive crises and cardiogenic shock unmasked by elective surgical procedures [11]. In such perioperative situations, acute intra-tumor hemorrhagic necrosis may precipitate catecholamine release, thereby inducing acute cardiogenic shock. Four patients had flu-like symptoms (fever, cough, myalgia) and acute heart failure with LV dysfunction and pulmonary edema, suggestive of myocarditis [13]. This particular clinical picture of pheochromocytoma mimicking fulminant myocarditis has been described previously [14] and should be known to physicians.

For our patients, pheochromocytoma was diagnosed based on abdominal CT or ultrasonography and then confirmed by elevated urinary catecholamines. Pheochromocytoma diagnosis usually relies on measuring plasma or urine metanephrines. However, cardiogenic shock and hypertensive crisis can physiologically increase catecholamine release and these patients frequently receive adrenaline or noradrenaline infusions. For those two reasons, metanephrine levels cannot be used in this setting to diagnose pheochromocytoma. As Amar and Eisenhofer suggested, this situation requires immediate imaging studies to search for a pheochromocytoma without prior biochemical evidence of a catecholamine-producing tumor [15].

A difficult issue is the optimal timing of pheochromocytoma excision. In a recent retrospective cohort study and literature review, emergency surgery during hypertensive crisis was associated with higher mortality and morbidity [16]. Stabilization with α-blockade prior to elective surgery was associated with shorter hospital stays and fewer postoperative complications. It seems useful to minimize the risk of catecholamine-induced hemodynamic fluctuations during anesthesia or pre-ablation tumor mobilization during surgery. Calcium channel blockers may be added, if necessary.

In our case series, α-blockers were gradually introduced in hemodynamically stabilized patients, followed by β-blockers to prevent tachycardia once cardiac systolic function had recovered and ECMO had been removed.

Five survivors underwent adrenalectomy 3 weeks to 4 months after the initial emergency presentation; the sixth pheochromocytoma was diagnosed 1 year later and removed under stable conditions and without perioperative complications. Celioscopic adrenalectomy was achievable in the six patients once heart function had fully recovered. None of these patients experienced another severe hypertensive crisis on α- and β-blockers before surgery. All six have been asymptomatic since the intervention.

As compared to previously reported cases, we described nine consecutive patients whereas most reported cases were single cases. Our patients were globally similar to previously reported cases: They shared same clinical characteristics and same short duration of ECMO support, and most adrenalectomy procedures were delayed after myocardial recovery. Contrasting with the good outcome (37/40 patients survived) of the previously reported patients, three of our nine patients died. This could be explained by publication bias related to single case reports; authors report most often successes than failures. Clinicians should be aware of this rare but curable cause of cardiogenic shock; it can be suspected in patients with cardiogenic shock associated with hypertension and easily confirmed using abdominal imaging. Moreover, we describe here a homogenous and successful single-center strategy for pheochromocytoma-induced cardiogenic shock management from the initial critical presentation to the scheduled adrenalectomy after heart recovery.

## Conclusion

Pheochromocytoma should systematically be considered for patients with Takotsubo cardiomyopathy, myocarditis, perioperative hypertensive crisis and/or unexplained cardiogenic shock. The definitive diagnosis is based on characteristic clinical features and abdominal imaging. For the most severe cases, ECMO support can be a life-saving therapy, allowing myocardial recovery within a few days. After hemodynamic stabilization, treatment should include α-blockade, with elective pheochromocytoma removal scheduled after myocardial recovery under stable conditions.
